# The relationship between depression symptoms and cortisol levels in adolescents: the role of somatic symptoms and cognitive function

**DOI:** 10.3389/fpsyt.2026.1850207

**Published:** 2026-06-24

**Authors:** Xia Lan, Pan Pan, Li Luo, Yu Li, Rongfang He, Hao Chen

**Affiliations:** 1 Department of Psychiatry, Luzhou Psychiatric Hospital, Luzhou, Sichuan, China; 2 Department of Psychiatry, Affiliated Hospital of Southwest Medical University, Luzhou, Sichuan, China

**Keywords:** adolescence, cognitive function, cortisol, depression, somatic symptoms

## Abstract

**Background:**

The relationship between clinical features of depression and hypothalamic-pituitary-adrenal (HPA) axis activity in adolescents remains poorly understood. This study examined the main and interactive effects of depressive severity, cognitive function, and somatic symptoms on cortisol levels in adolescent depression.

**Methods:**

This cross-sectional study included 138 adolescents. Based on their scores on the 24-item Hamilton Depression Rating Scale (HAMD-24) and the Patient Health Questionnaire-15 (PHQ-15), participants were divided into three groups: the depression with somatic symptoms group (DP group), the depression without somatic symptoms group (DW group), and a normal control group. To examine the main and interactive associations of clinical variables on cortisol levels, a general linear model (GLM) and simple slope analysis were employed.

**Results:**

Although bivariate correlations and group comparisons revealed no direct associations between cortisol levels and clinical indicators, the GLM identified significant interaction effects: PHQ−15 × HAMD−24 (*B* = 0.01, *p* < 0.05) and PHQ−15 × MoCA (*B* = 0.06, *p* < 0.05). The subsequent moderation model reached overall significance, further confirming the presence of these interactions (*F* = 2.331, *p* = 0.023, *R²* = 0.13). Simple slope analyses showed that higher somatic symptoms were significantly associated with lower cortisol only when depression severity was low−to−moderate and cognitive function was low, whereas a non−significant positive trend was observed when both were high.

**Conclusion:**

The association between somatic symptoms and cortisol in adolescents is differentially moderated by depression severity and cognitive function: a significant negative association was observed under conditions of low−to−moderate depression and low cognitive function, whereas a non−significant positive trend appeared when both were high. These hypothesis−generating findings require replication in larger, prospective studies.

## 1 Introduction

Adolescent depression presents a major global public health issue that impacts the mental and physical health of countless adolescents (1). In addition to primary symptoms such as ongoing sadness and a diminished interest in activities, depression can adversely affect cognitive abilities and trigger physical symptoms, ultimately hindering academic success, social relationships, and the overall standard of living (2). The dysregulation of the hypothalamic-pituitary-adrenal (HPA) axis is thought to be pivotal in understanding the pathophysiology of depression (3), and it is acknowledged as a key biological marker in depression studies (4). Evidence indicates that prolonged stress and increased cortisol levels may render individuals more susceptible to immune system declines and a range of physical issues associated with inflammation, which could worsen depressive symptoms (5–7). Nonetheless, most research investigating cortisol as a biological risk factor for depression has primarily concentrated on adult populations (8, 9). While an increasing number of studies propose that similar mechanisms may also be relevant for children and adolescents (10), the specific association of cortisol on the onset of major depressive disorder (MDD) in adolescents and young adults remains inadequately understood. Thus, while the directionality of these relationships has not been firmly established, a bidirectional or heterogeneous link between depressive characteristics and cortisol levels is plausible.

The diagnosis of major depressive disorder may include several clinical presentations that exhibit different phenomenological features, underlying biological processes, and responses to treatment (11). In adolescents, MDD often presents alongside physical complaints and mild cognitive deficits; however, the neuroendocrine factors associated with this clinical intricacy are not yet fully understood (12, 13). Alterations in the hypothalamic-pituitary-adrenal (HPA) system among teenagers suffering from severe depression are not consistently observed (10, 14). Preliminary research indicates that the association between depression, heightened HPA axis activity, and inflammation may primarily related to both physical and cognitive-affective symptoms (15–18). Consequently, increased inflammation and neuroendocrine activities may help in understanding the pathophysiology of the physical and cognitive aspects of depression (16, 19). While some investigations have indicated that elevated cortisol levels correspond with physical symptoms and reduced cognitive performance, there remain notable individual variations (20, 21). To date, however, few studies have examined how somatic symptoms and cognitive processes concurrently interact with depressive severity in relation to cortisol levels in adolescents.

Therefore, the present study aims to examine the associations between plasma cortisol levels and depressive severity, cognitive function, and somatic symptoms in adolescents with depression. Specifically, using a general linear model (GLM) and simple slope analysis (interaction model), we investigate whether somatic symptoms interact with depressive severity or cognitive function in their association with cortisol. By descriptively grouping patients with depression according to the presence or absence of clinically significant somatic symptoms, this study seeks to develop a more thorough insight into the pathophysiological mechanisms related to adolescent depression, thereby offering a theoretical foundation for accurate diagnosis and individualized treatment approaches. We hypothesize that plasma cortisol levels will differ between adolescents with depression and healthy individuals; that depressive severity, cognitive abilities, and physical symptoms will be associated with cortisol levels; and that the factors associated with plasma cortisol levels will differ among these descriptive groups.

## 2 Methods

### 2.1 Participants

**Figure 1:**
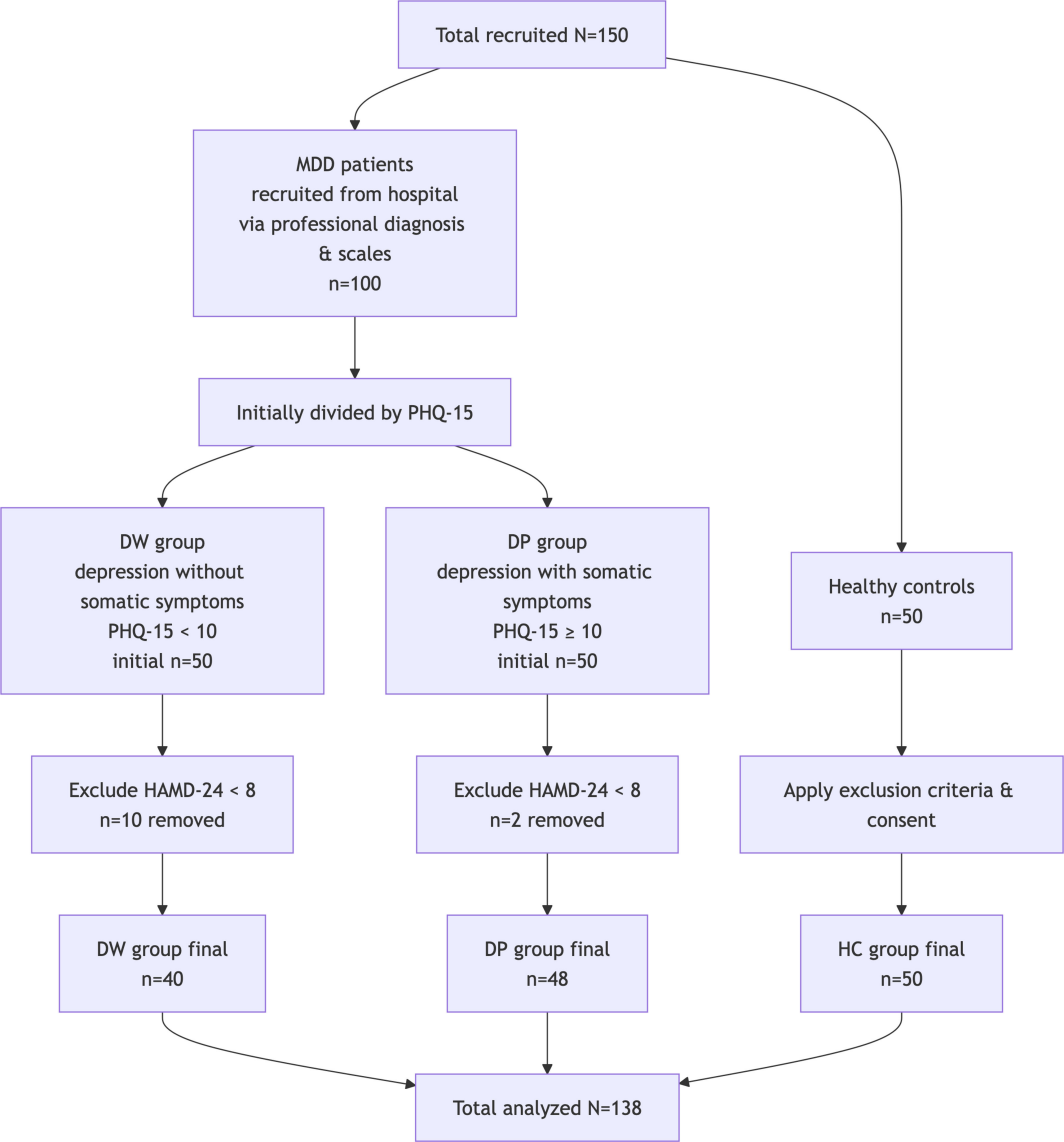
Flow diagram of participant selection.

100 adolescent MDD and 50 healthy controls were enrolled from the outpatient and inpatient psychiatric units of the Affiliated Hospital of Southwest Medical University and Luzhou Psychiatric Hospital. After screening based on exclusion criteria, 138 individuals were retained (see **Figure 1** for the flowchart). The recruitment process was assisted by qualified psychiatrists. Participants with MDD were recruited with the assistance of licensed psychiatrists based on the following inclusion criteria: (1) patients were preliminarily assessed by the Mini International Neuropsychiatric Interview (MINI) (22) and met the DSM-5 diagnostic criteria for MDD (23); (2) patients were experiencing their first depressive episode and had not received any systemic treatment involving antidepressants or antipsychotics; participants were therefore medication−naïve for these drug classes, but prior or concurrent psychotherapy was not an exclusion criterion. (3) age ranging from 10 to 19 years old, regardless of sex; (4) absence of specialized dietary requirements, tobacco use, and alcohol abuse habits.

Exclusion criteria included: (1) previous or current manic episodes, schizophrenia, or other psychiatric disorders; (2) prior treatment for depression, including psychotherapy, psychotropic medications, electroconvulsive therapy, and other interventions; (3) history of brain trauma, substance abuse, significant physical ailments (e.g., hypertension, coronary heart disease, metabolic diseases, asthma), severe periodontal (gum) diseases, dental abscess, or tooth injuries; (4) recent use of antibiotics or microecological modifiers within the past two months; and (5) special dietary requirements.

Following the quality control, the HC group included 50 adolescents recruited from schools in the vicinity of the Affiliated Hospital of Southwest Medical University. Inclusion criteria were as follows: (1) age ranging from 10 to 19 years, regardless of sex; (2) no history of prior psychiatric conditions or significant physical illnesses; (3) absence of specialized dietary requirements, tobacco use, and alcohol abuse habits. Exclusion criteria included: (1) history of head trauma, substance abuse, or severe physical illness; (2) specialized dietary requirements.

All adolescent participants signed the assent form, and their legal guardians signed the informed consent form. This study has received ethical approval from the Southwest Medical University Affiliated Hospital and the Luzhou Psychiatric Hospital located in Luzhou, Sichuan Province, China.

### 2.2 Instruments

#### 2.2.1 Hamilton depression rating scale

In this study, the Hamilton Depression Rating Scale-24 was utilized to evaluate symptoms of depression. This scale comprises 24 items divided into seven categories, including anxiety/somatization, weight, cognitive impairment, and others. Each component is rated from 0 to 4. The HAMD-24 score helps to ascertain the intensity of depressive symptoms: a total score of 8 or above indicates the presence of depression, with increasing scores reflecting more severe depressive conditions. For the assessment, a validated Chinese adaptation of the 24-item Hamilton Depression Rating Scale was employed. This particular version has been established for the Chinese population, showing a sensitivity of 0.87 and a specificity of 0.926 (24). In this sample, Cronbach’s α was found to be 0.851.

#### 2.2.2 Montreal cognitive assessment

To assess overall cognitive ability, the Montreal Cognitive Assessment (MoCA) was utilized, measuring various domains such as attention, concentration, executive functioning, memory, language, visuospatial abilities, abstract reasoning, and orientation (25). The highest possible score on the MoCA is 30; greater scores reflect better cognitive performance. The use of the MoCA for this non-commercial, academic research is permitted under the official policy (https://mocacognition.com/permission), and we hereby acknowledge its use. In this study, the Cronbach’s α was determined to be 0.748.

#### 2.2.3 Patient health questionnaire-15

Somatization was evaluated through the self-administered 15-item Patient Health Questionnaire (26). The PHQ-15 is commonly employed as a screening tool for somatization across various healthcare environments. This phenomenon represents one of the prevalent challenges within healthcare services, linked to significant functional disability and increased healthcare usage. The items in the PHQ-15 evaluate the occurrence of one or more somatic symptoms that cause distress (for example, fatigue, pain, and gastrointestinal issues) (26). Participants rated the intensity of 15 symptoms using a scale from 0 (“not bothered at all”) to 1 (“bothered a little”) or 2 (“bothered a lot”) over the preceding 4 weeks. According to the research of Kroenke et al., scores of 5, 10, and 15 represent validated cutoff points for low, medium, and high somatic symptom severity, respectively (26). Therefore, a cutoff of ≥10 was selected to define the depression with somatic symptoms (DP) group. This threshold is widely considered to indicate moderate to severe somatic symptom burden. In this study, the Cronbach’s α coefficient was found to be 0.94.

### 2.3 Cortisol determination

Samples of blood were gathered from individuals who had fasted in the morning hours, specifically from 06:00 to 08:00. The measurement of serum cortisol levels was performed quantitatively through chemiluminescence immunoassay (CLIA) using the Antuo A2000 analyzer provided by Zhengzhou Antuo Biotechnology Co., Ltd. To maintain the accuracy and dependability of the results, all samples underwent analysis in a single laboratory.

### 2.4 Procedure

The research was carried out in the following manner: (1) recruiting participants and securing informed consent; (2) conducting clinical interviews with psychiatrists to determine eligibility according to inclusion criteria; (3) administering the HAMD-24, MoCA, and PHQ-15 scales to those who met the eligibility requirements; (4) collecting fasting venous blood samples in the morning for the measurement of plasma cortisol levels; (5) gathering demographic information and medical history; and (6) processing the data and performing statistical analysis.

### 2.5 Statistical analysis

The data analysis was conducted with the help of SPSS Statistics version 26.0 alongside the PROCESS macro v4.2. Normality and homogeneity of variance were assessed for the data. Continuous variables were expressed as mean (standard deviation), and pairwise comparisons were conducted using Welch’s ANOVA and independent sample t-tests. Frequency (percentage) was used to present categorical data, with the chi-square test facilitating comparisons between the groups. To investigate correlations, Spearman correlation analyses were employed because the clinical variables showed non−normal distributions. Sex was coded (female = 0, male = 1).

A general linear model (GLM) was then employed to assess the independent influences of depression severity, cognitive abilities, and somatic symptoms on plasma cortisol levels, as well as to investigate both main and interaction effects. Covariates were age, sex, and illness duration (months from first symptom onset to enrolment). The group variable was not included in the final model because preliminary analysis showed high collinearity with the continuous symptom scores (VIF = 8.12). All continuous predictors were mean−centred before creating interaction terms. Variance inflation factors (VIF) for the final GLM were calculated using linear regression with collinearity diagnostics. To further investigate significant interactions, Hayes’ PROCESS Model 2 (27) was applied with the same specification: PHQ−15 as the independent variable, cortisol as the dependent variable, HAMD−24 and MoCA as simultaneous moderators, and age, sex, and illness duration as covariates, so that the PROCESS model corresponds exactly to the GLM. Sensitivity analyses were conducted to evaluate the robustness of the significant interactions. A p-value of less than 0.05 was deemed statistically significant.

## 3 Results

Based on the predefined exclusion criteria, 10 patients in the MDD without somatic symptoms group (DW) with HAMD-24 scores below 8 and 2 patients in the depression with somatic symptoms group (DP) were excluded. A total of 138 participants were therefore included in the final analysis. As shown in **Table 1**, the three groups (DW, DP, and HC) differed significantly in all clinical measures, including HAMD-24, PHQ-15, and MoCA scores (all *p* < 0.001). *Post-hoc* pairwise comparisons revealed that the severity of depression and somatic symptoms in the DP group was significantly higher than that in the DW group and the HC group (all comparisons significant). For cortisol, no post−hoc comparisons were performed because the overall group difference was not significant (*p* > 0.05). Although the sex distribution across groups did not differ significantly (*χ²* = 3.678, *p* > 0.05), the DP group had a numerically higher proportion of females (81.2%) than the HC (64%) and DW (70%) groups. Therefore, sex was included as a covariate to adjust for any potential confounding factors.

**Table 1:** Subject demographics.

Variable	HC (n=50)	DW (n=40)	DP (n=48)	Group differences	
				*H, χ2, F*	*p*
Age	18.54 (0.68)	15.63 (2.18)	15.65 (1.93)	*F* = 0.1	>0.05
Sex				*χ2* = 3.678	>0.05
female	32 (64)	28 (70)	39 (81.2)		
male	18 (36)	12 (30)	9 (18.8)		
Education	8.5 (0.25)	8.63 (1.18)	8.65 (1.33)	*F* = 0.31	>0.05
Illness duration (months)	0	12.85 (7.26)	13.96 (7.07)	*t* = 0.72^a^	>0.05
HAMD-24	4.74 (6.26)	14.83 (5.49)	20.44 (8.43)	*F* = 88.56	<0.001
MoCA	28.4 (2.15)	23.18 (4.21)	24.31 (2.48)	*F* = 79.65	<0.001
PHQ-15	3.44 (3.6)	2.5 (1.32)	18.33 (3.63)	*F* = 79.32	<0.001
Cortisol (μg/dl)	10.74 (3.92)	12.23 (6.18)	12.12 (5.78)	*F* = 0.366	>0.05

HC, Healthy controls; DW, the depression without somatic symptoms group; DP, the depression with somatic symptoms group; HAMD-24, 24-item Hamilton Depression Rating Scale; PHQ-15, the Patient Health Questionnaire-15; MoCA, Montreal Cognitive Assessment. ^a^ Illness duration was compared only between DW and DP using independent−samples t−test.

**Table 2** presents the Spearman correlation analyses. Depression severity (HAMD-24) was positively correlated with somatic symptom severity (PHQ-15) and illness duration (*rs* = 0.575 to 0.618), and negatively correlated with cognitive function (MoCA), age, and sex (*rs* = −0.437 to −0.175). In contrast, cognitive function was negatively correlated with somatic symptoms and illness duration (*rs* = −0.359 to −0.184), but positively correlated with age (*rs* = 0.687). Sex was also significantly associated with both depression severity and somatic symptom burden (*rs* = −0.437 to −0.314). Consistent with the group comparisons, cortisol levels were not significantly correlated with HAMD-24, PHQ-15, or MoCA scores in the overall sample; however, a weak positive correlation was observed between cortisol levels and illness duration (*rs* = 0.169).

**Table 2:** Bivariate correlations between study variables.

Variable	M	SD	HAMD-24	MoCA	PHQ-15	Cortisol	Sex	Age
HAMD-24	13.12	9.6						
MoCA	25.46	3.73	−0.413***					
PHQ-15	8.35	7.96	0.618***	−0.184*				
Cortisol	11.65	5.32	0.114	−0.129	0.037			
Sex ^a^	0.28	0.45	−0.175*	0.103	−0.206*	0.149		
Age	16.69	2.18	−0.437***	0.687***	−0.314***	0.012	0.179*	
Illness duration	8.58	8.63	0.575***	−0.359***	0.397***	0.169*	−0.048	−0.194*

M represents means and SD represents standard deviations calculated from the pooled datasets. ^a^ Sex (female = 0, male = 1). HAMD-24, 24-item Hamilton Depression Rating Scale; PHQ-15, the Patient Health Questionnaire-15; MoCA, Montreal Cognitive Assessment. *indicates *p* < 0.05; **indicates *p* < 0.01; ***indicates *p* < 0.001.

To further examine the effects of depression severity, cognitive function, and somatic symptoms on cortisol levels, a general linear model was fitted with age, sex, and illness duration as covariates. As shown in **Table 3**, sex showed a significant main effect (*B* = 2.12, *p* < 0.05). In addition, significant interaction effects were identified between depression severity and somatic symptoms (*B* = 0.01, *p* < 0.05), as well as between cognitive function and somatic symptoms (*B* = 0.06, *p* < 0.05). Variance inflation factors (VIF) for all predictors in the final model ranged from 1.59 to 2.65 (**Table 3**), well below the conventional threshold of 5 or 10, indicating no problematic multicollinearity (28). Furthermore, we tested the robustness of the joint model by examining each interaction term separately; neither was significant when tested alone (**Supplementary Table 1**), consistent with our theoretical framework that somatic symptoms influence cortisol only through the simultaneous moderation of both depression severity and cognitive function. Given the exploratory nature of the study, these findings should be interpreted as hypothesis−generating and require replication in larger samples. To further clarify these interaction effects, moderation and simple slope analyses were conducted to evaluate the association between PHQ-15 scores and cortisol at low (mean − 1SD), mean, and high (mean + 1SD) levels of each moderator.

**Table 3:** The results of a general linear model for cortisol levels with depression severity, somatic symptoms, and cognitive function.

Source	B	SE	*95%CI*	VIF	Wald *χ2*	*P*
age	0.29	0.30	(−0.3, 0.87)	2.36	0.93	0.336
sex	2.12	0.98	(0.2, 4.03)	1.09	4.70	0.030*
Illness duration	0.10	0.07	(−0.03, 0.23)	1.84	2.31	0.129
HAMD-24	−0.01	0.07	(−0.14, 0.13)	2.54	0.01	0.926
MoCA	−0.13	0.18	(−0.49, 0.23)	2.65	0.51	0.475
PHQ-15	0.01	0.08	(−0.14, 0.16)	2.01	0.03	0.872
HAMD-24*MoCA	−0.02	0.02	(−0.06, 0.03)	2.14	0.48	0.490
MoCA*PHQ-15	0.06	0.02	(0.01, 0.10)	2.33	5.16	0.023*
HAMD-24*PHQ-15	0.01	0.01	(0, 0.03)	1.59	3.99	0.046*
Intercept	10.51	0.61	(9.31, 11.71)		293.21	0.000

HC, Healthy controls; DW, the depression without somatic symptoms group; DP, the depression with somatic symptoms group; HAMD-24, 24-item Hamilton Depression Rating Scale; PHQ-15, the Patient Health Questionnaire-15; MoCA, Montreal Cognitive Assessment; VIF, variance inflation factor. All continuous predictors were mean−centred before creating interaction terms. * indicates *p* < 0.05.

**Figure 2:**
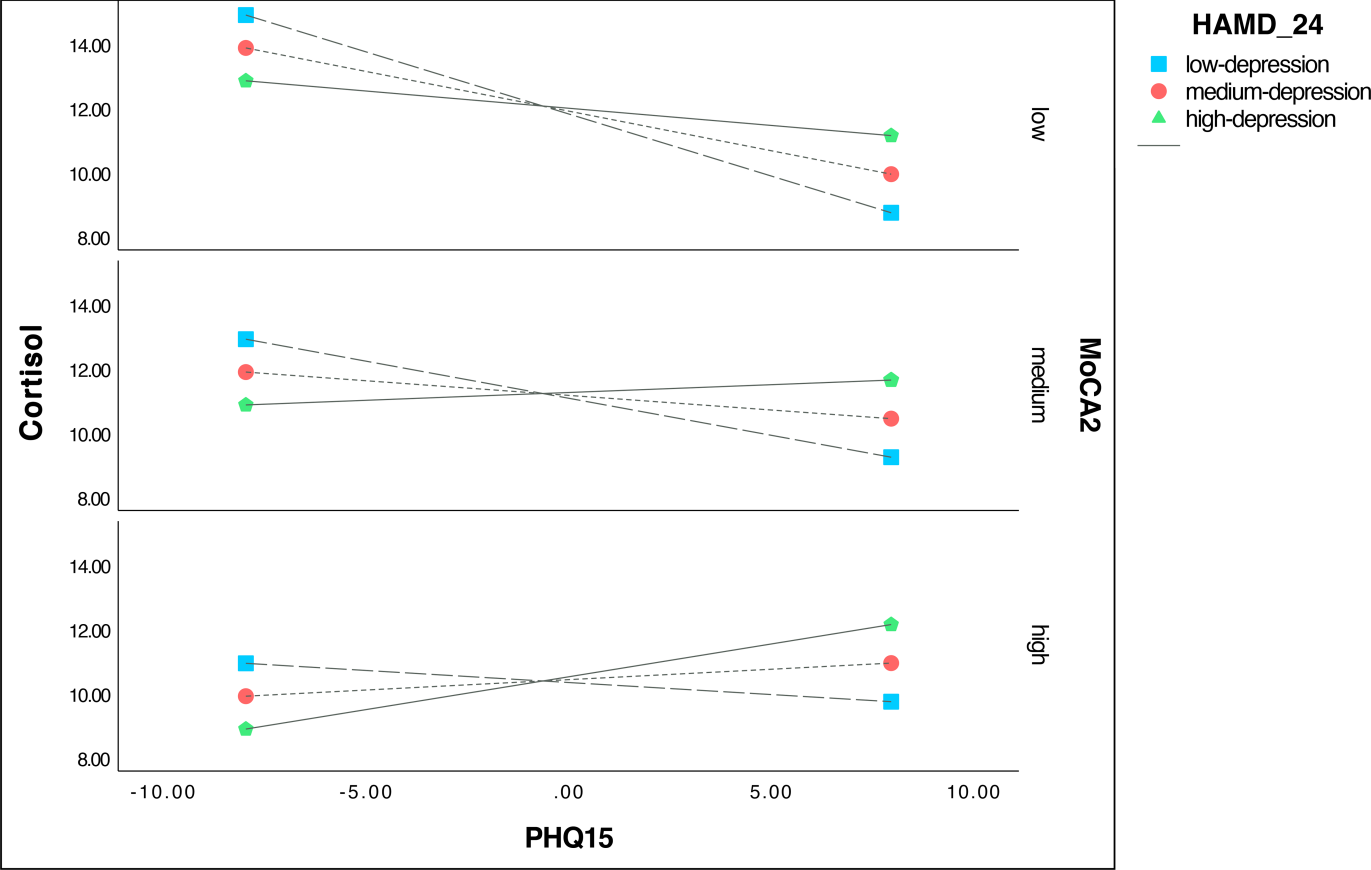
The moderating effects of depression and cognition on the association between somatic symptoms and cortisol levels. The PHQ-15 score has undergone centralization and standardization processing (z-score) for adjustment analysis; therefore, the x−axis range represents standard deviation units. Low, medium, and high levels of HAMD−24 and MoCA correspond to mean–1 SD, mean, and mean+1 SD, respectively.

The moderation model was statistically significant (*F* = 2.331, *p* = 0.023) and explained approximately 13% of the variance in cortisol levels (*R²* = 0.13). Consistent with the GLM findings, both interaction terms remained significant (**Supplementary Table 2**; **Figure 2**). Simple slope analyses further indicated that the association between somatic symptoms and cortisol varied according to both depression severity and cognitive function. As shown in **Supplementary Table 3**, when HAMD-24 was low and MoCA was low, PHQ-15 was significantly negatively correlated with cortisol (*B* = −0.386, *p* = 0.017); similarly, when HAMD-24 was at its mean and MoCA was low, PHQ-15 was also significantly negatively correlated with cortisol (*B* = −0.247, *p* = 0.041). At high HAMD-24 levels, however, a positive trend emerged when MoCA was high (*B* = 0.204), whereas the association remained negative when MoCA was low (*B* = −0.107); although neither reached statistical significance, these patterns are illustrated in **Supplementary Figure 1**.

## 4 Discussion

This research examined the intricate relationships among depression severity, cognitive abilities, physical symptoms, and cortisol levels in adolescents. The findings provided new insights, suggesting that somatic symptoms in this age group may be associated with cortisol levels under specific conditions, namely, depending on the severity of depression and cognitive function. However, this association was significantly influenced by cognitive abilities and the severity of depression. Furthermore, within the adolescent population, the relationship between physical symptoms and HPA axis activity was not straightforward; rather, it was shaped by the severity of both cognitive function and depression. Collectively, these factors were essential for understanding physiological responses. A comprehensive understanding of these complex relationships was crucial for developing more targeted intervention and treatment strategies for this population.

A key discovery of this study was that the relationship between somatic symptoms and cortisol levels was not direct; rather, it was influenced by the interdependent moderation of depression severity and cognitive function. While bivariate correlations and group comparisons did not reveal a direct link, the general linear model identified significant interactions, indicating that if cortisol was associated with depression, this relationship arose from a complex interplay rather than a straightforward main effect. Specifically, in cases of mild depression and lower cognitive function, a significant negative correlation was observed between physical symptoms and cortisol. This is consistent with the low cortisol phenomenon observed in stress-related physical disorders (29, 30). This pattern has been reported across chronic fatigue syndrome, fibromyalgia, and somatoform disorders, where lower cortisol levels are frequently observed despite significant physical complaints (29, 31, 32). For instance, moderate physical stress seems to have beneficial effects, such as enhancing cognitive functions (33–35), as well as larger prefrontal cortex and lower cortisol levels (36, 37). Furthermore, the absence of a significant negative association when cognitive function was higher aligns with evidence that better cognitive abilities may enhance interoceptive awareness and physiological stress reactivity, potentially masking the hypocortisol pattern (38, 39). Thus, the negative somatic symptom−cortisol link appears most evident in adolescents with relatively mild depression and poorer cognitive function, a finding that underscores the importance of considering both affective and cognitive contexts when interpreting neuroendocrine correlates of somatic complaints.

The significant PHQ−15×HAMD−24 interaction further revealed a clinically informative pattern when examined alongside cognitive function. By contrast, although not statistically significant, a positive trend emerged in adolescents with high depression severity and high cognitive function, such that higher somatic symptoms tended to be associated with higher cortisol levels. This shift from a significant negative association (under low−to−moderate depression and low cognition) to a non−significant positive trend (under high depression and high cognition) suggests a potential continuum of HPA axis reactivity that may parallel the well−recognised cortisol profiles of melancholic (40, 41) versus atypical depression (14). Adolescents with severe depression and preserved cognitive abilities may experience a more agitated, hyper−aroused state, leading to heightened HPA−axis activity and consequently higher cortisol when somatic symptoms are present (40, 41). This interpretation is supported by evidence that higher cognitive function can amplify threat detection and physiological stress responses, particularly in individuals with severe affective disturbances (38, 39). In contrast, those with milder depression and lower cognitive function may exhibit a more passive, fatigue−related phenotype with HPA blunting (29, 30). Nonetheless, given the exploratory nature and modest sample size, these speculative findings require replication in larger longitudinal studies with repeated cortisol sampling.

The relationship between physical symptoms, cognitive function, and cortisol levels provides valuable insights into the interplay of psychological, physiological, and cognitive processes. Specifically, a significant negative association was observed only when cognitive function was low, which is similar to previous studies (42). One possible explanation is that individuals with lower cognitive function may underreport somatic symptoms due to differences in interoceptive awareness, reporting style, or alexithymia, which could also influence the observed association. In such cases, cognitive processing of physical discomfort may be less pronounced, potentially leading to reduced activation of the HPA axis (43, 44). Conversely, enhanced cognitive abilities may increase awareness of bodily sensations and heighten physiological stress reactivity (45, 46), potentially masking the negative cortisol−somatic symptom link and even contributing to a positive trend. These findings underscored the importance of cognitive processing in understanding depressive symptoms and their physiological correlates, particularly in adolescents facing cognitive challenges and a high somatic burden. Given the self−report nature and lack of direct bivariate associations, these speculative findings need replication with objective measures and repeated cortisol sampling.

The presented bivariate correlations provided essential contextual insights. A robust positive correlation was found between HAMD-24 and PHQ-15, highlighting the significant overlap of cognitive-affective symptoms with somatic symptoms in cases of depression (47, 48). The inverse relationship observed between the severity of depression and cognitive function aligned with previous research indicating cognitive deficits as a key characteristic of depression (49). Notably, it was reported that female adolescents tend to score higher on assessments of depression and somatic symptoms, consistent with epidemiological studies showing a greater incidence of depression and somatization in adolescent females (50, 51). Furthermore, a modest yet meaningful positive correlation was identified between disease duration and cortisol levels, despite the lack of a direct link to symptom scores across the general population. This finding suggested that the chronic nature of the illness may exert a persistent, subtle influence on HPA axis activity (52). This insight enhanced the understanding of how long-term depressive states might affect physiological processes in the body, potentially informing future research and clinical approaches to managing chronic depression.

It is crucial to acknowledge the limitations of this study. Firstly, the cross-sectional design precludes causal inference, and the relatively small sample size limits both statistical power and generalizability. Future studies should adopt longitudinal designs with larger samples. Secondly, relying on a single morning cortisol measurement oversimplifies the complexity of the HPA axis; subsequent research should collect repeated samples (e.g., diurnal patterns, cortisol awakening response) or implement challenge tests. Thirdly, the PHQ-15 is a self-report measure, and we did not collect BMI data. Self-reported symptoms may be influenced by reporting style, health anxiety, or alexithymia, and unmeasured body composition could confound the associations between cortisol and symptoms. Future studies should include objective measures of interoceptive accuracy, repeated cortisol sampling, and BMI as a covariate. Lastly, the exclusion criteria were more stringent for the MDD group than for healthy controls, which may introduce selection bias; future research should apply symmetrical medical screening to both groups.

This research highlighted the interconnected relationships among depression, somatic symptoms, cognitive function, and cortisol levels in adolescents. Our interaction models suggested that the severity of depression and cognitive ability may influence the relationship between somatic symptoms and cortisol. Specifically, a significant negative association was observed only when depression severity was low to moderate and cognitive function was low; a non−significant positive trend appeared when both depression severity and cognitive function were high. These findings are exploratory and hypothesis−generating, not confirmatory, given the absence of direct group differences or bivariate correlations. Replication in larger, prospective studies with repeated cortisol sampling is needed. Nevertheless, this study offers an interaction−based perspective that encourages future research to integrate symptom−specific and cognitive−contextual factors.

## Data Availability

The datasets presented in this article are not readily available due to patient confidentiality and ethical restrictions. Requests to access the datasets should be directed to the corresponding author.

## References

[1] Mariani N McLaughlin A Lambert E Kose M Nikkheslat N Patsalos O . Disentangling the effects of depression and perceived stress on cortisol levels in individuals with obesity: Preliminary results from a cross-sectional study. Psychoneuroendocrinology. (2023) 158:106387. doi: 10.1016/j.psyneuen.2023.106387 37801751

[2] Chung J Mukerji S Kozlowska K . Cortisol and alpha-amylase awakening response in children and adolescents with functional neurological (conversion) disorder. Aust N Z J Psychiatry. (2023) 57:115–29. doi: 10.1177/00048674221082520 35297291

[3] Lee BH Kim YK . The roles of BDNF in the pathophysiology of major depression and in antidepressant treatment. Psychiatry Investig. (2010) 7:231–5. doi: 10.4306/pi.2010.7.4.231 21253405 PMC3022308

[4] Wexler J Ajibewa TA Lee J Toledo-Corral CC Hasson RE . Community violence exposure and cortisol awakening responses in adolescents who are overweight/obese. Psychoneuroendocrinology. (2020) 121:104842. doi: 10.1016/j.psyneuen.2020.104842 32892064

[5] Lucassen EA Cizza G . The hypothalamic-pituitary-adrenal axis, obesity, and chronic stress exposure: Sleep and the HPA axis in obesity. Curr Obes Rep. (2012) 1:208–15. doi: 10.1007/s13679-012-0028-5 23162786 PMC3498460

[6] Keller J Gomez R Williams G Lembke A Lazzeroni L Murphy GM . HPA axis in major depression: cortisol, clinical symptomatology and genetic variation predict cognition. Mol Psychiatry. (2017) 22:527–36. doi: 10.1038/mp.2016.120 27528460 PMC5313380

[7] Chaddha A Robinson EA Kline-Rogers EK Alexandris-Souphis TA Rubenfire M . Mental health and cardiovascular disease. Am J Med. (2016) 129:1145–8. doi: 10.1016/j.amjmed.2016.05.018 27288855

[8] Kennis M Gerritsen L van Dalen M Williams A Cuijpers P Bockting C . Prospective biomarkers of major depressive disorder: a systematic review and meta-analysis. Mol Psychiatry. (2020) 25:321–38. doi: 10.1038/s41380-019-0585-z 31745238 PMC6974432

[9] Zorn JV Schür RR Boks MP Kahn RS Joëls M Vinkers CHV . Cortisol stress reactivity across psychiatric disorders: A systematic review and meta-analysis. Psychoneuroendocrinology. (2017) 77:25–36. doi: 10.1016/j.psyneuen.2016.11.036 28012291

[10] Lopez-Duran NL Kovacs M George CJ . Hypothalamic-pituitary-adrenal axis dysregulation in depressed children and adolescents: a meta-analysis. Psychoneuroendocrinology. (2009) 34:1272–83. doi: 10.1016/j.psyneuen.2009.03.016 19406581 PMC2796553

[11] Halbreich U . Major depression is not a diagnosis, it is a departure point to differential diagnosis—clinical and hormonal considerations:(A commentary and elaboration on antonejevic's paper). Psychoneuroendocrinology. (2006) 31:16–22. doi: 10.1016/j.psyneuen.2005.06.004 16242851

[12] Avery JA Drevets WC Moseman SE Bodurka J Barcalow JC Simmons WK . Major depressive disorder is associated with abnormal interoceptive activity and functional connectivity in the insula. Biol Psychiatry. (2014) 76:258–66. doi: 10.1016/j.biopsych.2013.11.027 24387823 PMC4048794

[13] Lepine JP Briley M . The epidemiology of pain in depression. Hum Psychopharmacol. (2004) 19 Suppl 1:S3–7. doi: 10.1002/hup.618 15378670

[14] Stetler C Miller GE . Depression and hypothalamic-pituitary-adrenal activation: a quantitative summary of four decades of research. Psychosom Med. (2011) 73:114–26. doi: 10.1097/psy.0b013e31820ad12b 21257974

[15] White J Kivimäki M Jokela M Batty GD . Association of inflammation with specific symptoms of depression in a general population of older people: The English Longitudinal Study of Ageing. Brain Behav Immun. (2017) 61:27–30. doi: 10.1016/j.bbi.2016.08.012 27562420

[16] Jokela M Virtanen M Batty GD Kivimäki M . Inflammation and specific symptoms of depression. JAMA Psychiatry. (2016) 73:87–8. doi: 10.1001/jamapsychiatry.2015.1977 26579988

[17] Duivis HE Vogelzangs N Kupper N Jonge PD Penninx BWHJ . Differential association of somatic and cognitive symptoms of depression and anxiety with inflammation: findings from the Netherlands Study of Depression and Anxiety (NESDA). Psychoneuroendocrinology. (2013) 38:1573–85. doi: 10.1016/j.psyneuen.2013.01.002 23399050

[18] Bosch NM Riese H Dietrich A Ormel J Verhulst FC Oldehinkel AJ . Preadolescents' somatic and cognitive-affective depressive symptoms are differentially related to cardiac autonomic function and cortisol: the TRAILS study. Psychosom Med. (2009) 71:944–50. doi: 10.1097/psy.0b013e3181bc756b 19834052

[19] Chu AL Stochl J Lewis G Zammit S Jones PB Khandaker GM . Longitudinal association between inflammatory markers and specific symptoms of depression in a prospective birth cohort. Brain Behav Immun. (2019) 76:74–81. doi: 10.1016/j.bbi.2018.11.007 30414442 PMC6363967

[20] Lee BK Glass TA Wand GS McAtee MJ Bandeen-Roche K Bolla KI . Apolipoprotein e genotype, cortisol, and cognitive function in community-dwelling older adults. Am J Psychiatry. (2008) 165:1456–64. doi: 10.1176/appi.ajp.2008.07091532 18593777 PMC2579316

[21] Libin D Xiucui Z Weibing M . Correlation between cortisol level and physical symptomsin adolescents with major depressive disorder. Chongqing Med. (2022) 51:76–9. doi: 10.3969/j.issn.1671-8348.2022.01.016

[22] Sheehan DV Lecrubier Y Sheehan KH Amorim P Janavs J Weiller E . The Mini-International Neuropsychiatric Interview (MINI): the development and validation of a structured diagnostic psychiatric interview for DSM-IV and ICD-10. J Clin Psychiatry. (1998) 59:22–33. doi: 10.4088/JCP.v59n0105 9881538

[23] American Psychiatric Association . Diagnostic and Statistical Manual of Mental Disorders: DSM-5. Washington, DC: American Psychiatric Association (2013).

[24] Zheng YP Zhao JP Phillips M Liu JB Cai MF Sun SQ . Validity and reliability of the chinese hamilton depression rating scale. Br J Psychiatry. (1988) 152:660–4. doi: 10.1192/bjp.152.5.660 3167442

[25] Nasreddine ZS Phillips NA Bédirian V Charbonneau S Whitehead V Collin I . The Montreal Cognitive Assessment, MoCA: a brief screening tool for mild cognitive impairment. J Am Geriatr Soc. (2005) 53:695–9. doi: 10.1111/j.1532-5415.2005.53221.x 15817019

[26] Kroenke K Spitzer RL Williams JB . The PHQ-15: validity of a new measure for evaluating the severity of somatic symptoms. Psychosom Med. (2002) 64:258–66. doi: 10.1097/00006842-200203000-00008 11914441

[27] Hayes AF . PROCESS: A versatile computational tool for observed variable mediation, moderation, and conditional process modeling. In: University of Kansas, KS (2012). doi: 10.1108/978-1-62396-246-320251009

[28] O’Brien RM . A caution regarding rules of thumb for variance inflation factors. Qual Quant. (2007) 41:673–80. doi: 10.1007/s11135-006-9018-6

[29] Heim C Ehlert U Hellhammer DH . The potential role of hypocortisolism in the pathophysiology of stress-related bodily disorders. Psychoneuroendocrinology. (2000) 25:1–35. doi: 10.1016/s0306-4530(99)00035-9 10633533

[30] Tak LM Rosmalen JGM . Dysfunction of stress responsive systems as a risk factor for functional somatic syndromes. J Psychosomatic Res. (2010) 68:461–8. doi: 10.1016/j.jpsychores.2009.12.004 20403505

[31] Staufenbiel SM Penninx BWJH Spijker AT Elzinga BM van Rossum EFC . Hair cortisol, stress exposure, and mental health in humans: a systematic review. Psychoneuroendocrinology. (2013) 38:1220–35. doi: 10.1016/j.psyneuen.2012.11.015 23253896

[32] Tak LM Cleare AJ Ormel J Manoharan A Kok IC Wessely S . Meta-analysis and meta-regression of hypothalamic-pituitary-adrenal axis activity in functional somatic disorders. Biol Psychol. (2011) 87:183–94. doi: 10.1016/j.biopsycho.2011.02.002 21315796

[33] Parker KJ Buckmaster CL Justus KR Schatzberg AF Lyons DM . Mild early life stress enhances prefrontal-dependent response inhibition in monkeys. Biol Psychiatry. (2005) 57:848–55. doi: 10.1016/j.biopsych.2004.12.024 15820705

[34] Schwabe L Joëls M Roozendaal B Wolf OT Oitzl MS . Stress effects on memory: an update and integration. Neurosci Biobehav Rev. (2012) 36:1740–9. doi: 10.1016/j.neubiorev.2011.07.002 21771612

[35] Schwabe L Wolf OT Oitzl MS . Memory formation under stress: quantity and quality. Neurosci Biobehav Rev. (2010) 34:584–91. doi: 10.1016/j.neubiorev.2009.11.015 19931555

[36] Arnsten AF . Stress signalling pathways that impair prefrontal cortex structure and function. Nat Rev Neurosci. (2009) 10:410–22. doi: 10.1038/nrn2648 19455173 PMC2907136

[37] Lupien SJ McEwen BS Gunnar MR Heim C . Effects of stress throughout the lifespan on the brain, behaviour and cognition. Nat Rev Neurosci. (2009) 10:434–45. doi: 10.1038/nrn2639 19401723

[38] George MY Abdel Mageed SS Mansour DE Fawzi SF . The cortisol axis and psychiatric disorders: an updated review. Pharmacol Rep. (2025) 77:1573–99. doi: 10.1007/s43440-025-00782-x 40956392 PMC12647353

[39] Thayer JF Brosschot JF . Psychosomatics and psychopathology: looking up and down from the brain. Psychoneuroendocrinology. (2005) 30:1050–8. doi: 10.1016/j.psyneuen.2005.04.014 16005156

[40] Knorr U Vinberg M Kessing LV Wetterslev J . Salivary cortisol in depressed patients versus control persons: a systematic review and meta-analysis. Psychoneuroendocrinology. (2010) 35:1275–86. doi: 10.1016/j.psyneuen.2010.04.001 20447770

[41] Pariante CM Lightman SL . The HPA axis in major depression: classical theories and new developments. Trends Neurosci. (2008) 31:464–8. doi: 10.1016/j.tins.2008.06.006 18675469

[42] Ruiz-Robledillo N Gonzalez-Bono E Moya-Albiol L . Lack of institutional support entails disruption in cortisol awakening response in caregivers of people with high-functioning autism. J Health Psychol. (2014) 19:1586–96. doi: 10.1177/1359105313496444 23933951

[43] Takahashi T Higuchi Y Komori Y Nishiyama S Takayanagi Y Sasabayashi D . Pituitary volume and socio-cognitive functions in individuals at risk of psychosis and patients with schizophrenia. Front Psychiatry. (2018) 9:574. doi: 10.3389/fpsyt.2018.00574 30473669 PMC6237858

[44] Liu Y Gang X Gao Y Wang GX . Causal associations between congenital adrenal hyperplasia and neuropsychiatric conditions- a Mendelian randomization study. Endocrine. (2025) 89:291–307. doi: 10.1007/s12020-025-04237-4 40307628 PMC12227516

[45] Feola B Armstrong K Woodward ND Heckers S Blackford JU . Childhood temperament is associated with distress, anxiety and reduced quality of life in schizophrenia spectrum disorders. Psychiatry Res. (2019) 275:196–203. doi: 10.1016/j.psychres.2019.03.016 30925307 PMC6872191

[46] Castro-Ramirez F Paz-Pérez MA McGuire TC Rankin O García Alfaro MC Audirac AM . A qualitative examination of the impact of suicidal thoughts and behavior on help-seeking among university students in Colombia and Mexico. J Behav Cognit Ther. (2023) 33:67–80. doi: 10.1016/j.jbct.2023.04.001 37680902 PMC10482072

[47] Ehrmann D Krause-Steinrauf H Uschner D Wen H Hoogendoorn CJ Crespo-Ramos G . Differential associations of somatic and cognitive-affective symptoms of depression with inflammation and insulin resistance: cross-sectional and longitudinal results from the Emotional Distress Sub-Study of the GRADE study. Diabetologia. (2025) 68:1403–15. doi: 10.1007/s00125-025-06369-8 39951058 PMC12176517

[48] Brailean A Comijs HC Aartsen MJA Prince M Prina AM Beekman A . Late-life depression symptom dimensions and cognitive functioning in the Longitudinal Aging Study Amsterdam (LASA). J Affect Disord. (2016) 201:171–8. doi: 10.1016/j.jad.2016.05.027 27235820 PMC4914607

[49] Wiest A Maurer JJ Weber F Chung S . A hypothalamic circuit mechanism underlying the impact of stress on memory and sleep. bioRxiv. (2024). doi: 10.1101/2024.10.17.618467

[50] Zhou J Yuan XF Qi H Liu R Li YQ Huang HH . Prevalence of depression and its correlative factors among female adolescents in China during the coronavirus disease 2019 outbreak. Global Health. (2020) 16:69. doi: 10.1186/s12992-020-00601-3 32723373 PMC7385712

[51] Duberg A Jutengren G Hagberg L Möller MM . The effects of a dance intervention on somatic symptoms and emotional distress in adolescent girls: a randomized controlled trial. J Int Med Res. (2020) 48:300060520902610. doi: 10.1177/0300060520902610 32019389 PMC7111017

[52] Cui B Peng F Lu JX He B Su QT Luo HD . Cancer and stress: nextGen strategies. Brain Behav Immun. (2021) 93:368–83. doi: 10.1016/j.bbi.2020.11.005 33160090

