# Supplementary material for: The relationship between depression symptoms and cortisol levels in adolescents: the role of somatic symptoms and cognitive function
Source: Front Psychiatry. 2026 Jun 24;17:1850207. doi: 10.3389/fpsyt.2026.1850207 (PMC13343220; doi:10.3389/fpsyt.2026.1850207)
Supplement: Supplementary file 1 [file SupplementaryFile1.docx]

**Online Supplemental Appendix for**

**The Relationship Between Depression Symptoms and Cortisol Levels in Adolescents:**

**The Role of Somatic Symptoms and Cognitive Function**

**Authors’ note:**

We developed these materials to provide additional technical information and to keep the main manuscript from becoming needlessly long.

**Supplementary Figure 1.** The association between cognition, physical symptoms, and morning cortisol levels in adolescents across groups with varying degrees of depression.

Note: The PHQ-15 score has undergone centralization and standardization processing (z-score) for adjustment analysis; therefore, the x‑axis range represents standard deviation units. Low, medium, and high levels of HAMD‑24 and MoCA correspond to mean–1 SD, mean, and mean+1 SD, respectively.

**Supplementary Table 1.** Sensitivity analysis: Separate GLM models for each interaction.

| Variable | Model 1 (PHQ-15 × HAMD-24) | | | | Model 2 (PHQ-15 × MoCA) | | | |
| --- | --- | --- | --- | --- | --- | --- | --- | --- |
|  | B | SE | *95%CI* | *P* | B | SE | *95%CI* | *P* |
| **Main effects** |  |  |  |  |  |  |  |  |
| PHQ-15 | –0.052 | 0.073 | (–0.196,0.092) | 0.479 | 0.051 | 0.066 | (–0.078,0.179) | 0.44 |
| HAMD-24 | 0.032 | 0.071 | (–0.108,0.172) | 0.653 | — | — | — | — |
| MoCA | — | — | — | — | –0.222 | 0.176 | (–0.567,0.122) | 0.206 |
| **Interaction** |  |  |  |  |  |  |  |  |
| MoCA*PHQ-15 | — | — | — | — | 0.029 | 0.018 | (–0.005,0.064) | 0.099 |
| HAMD-24*PHQ-15 | 0.009 | 0.006 | (–0.003,0.021) | 0.162 | — | — | — | — |
| **Covariates** |  |  |  |  |  |  |  |  |
| age | 0.045 | 0.23 | (–0.405,0.495) | 0.844 | 0.403 | 0.289 | (–0.164,0.97) | 0.163 |
| sex | 1.93 | 0.998 | (–0.026,3.886) | 0.053 | 1.87 | 0.986 | (–0.062,3.801) | 0.058 |
| Illness duration | 0.107 | 0.064 | (20.604,33.03) |  | 0.072 | 0.059 | (–0.043,0.187) | 0.219 |

**Note:** Each model was run on the full sample (N=138, including healthy controls). The group variable was not included. Standard errors, 95% confidence intervals, and p‑values are based on robust estimation.

**Supplementary Table 2.** PROCESS macro (Model 2) analysis: Main and interaction effects of PHQ‑15, HAMD‑24, and MoCA on cortisol.

| **Variable** | **B** | **SE** | ***t*** | ***p*** | ***95% CI*** |
| --- | --- | --- | --- | --- | --- |
| HAMD‑24 | 0.009 | 0.068 | 0.135 | 0.893 | (–0.126, 0.144) |
| MoCA | –0.198 | 0.175 | –1.132 | 0.260 | (–0.544, 0.148) |
| PHQ‑15 | –0.091 | 0.106 | –0.860 | 0.391 | (–0.300, 0.118) |
| **PHQ‑15 × HAMD‑24** | **0.015** | **0.007** | **2.164** | **0.032** | **(0.001, 0.028)** |
| **PHQ‑15 × MoCA** | **0.042** | **0.019** | **2.149** | **0.034** | **(0.003, 0.080)** |

**Note:** PROCESS macro (Hayes, 2018), Model 2. Statistically significant interaction terms (p < 0.05) are shown in bold. 95% CI = bootstrap confidence interval (5,000 resamples).

**Supplementary Table 3.** Conditional effects of PHQ-15 on cortisol at specific values of HAMD-24 and MoCA.

| **Condition** | **Effect** | **SE** | ***t*** | ***p*** | ***95% CI*** |
| --- | --- | --- | --- | --- | --- |
| Low HAMD, Low MoCA | –0.386 | 0.159 | –2.425 | 0.017 | (–0.702, –0.071) |
| Low HAMD, Mean MoCA | –0.231 | 0.138 | –1.672 | 0.097 | (–0.504, 0.042) |
| Low HAMD, High MoCA | –0.075 | 0.152 | –0.492 | 0.623 | (–0.376, 0.226) |
| Mean HAMD, Low MoCA | –0.247 | 0.119 | –2.068 | 0.041 | (–0.483, –0.011) |
| Mean HAMD, Mean MoCA | –0.091 | 0.106 | –0.860 | 0.391 | (–0.300, 0.118) |
| Mean HAMD, High MoCA | 0.065 | 0.137 | 0.474 | 0.637 | (–0.206, 0.335) |
| High HAMD, Low MoCA | –0.107 | 0.107 | –1.002 | 0.318 | (–0.319, 0.104) |
| High HAMD, Mean MoCA | 0.049 | 0.108 | 0.450 | 0.654 | (–0.165, 0.262) |
| High HAMD, High MoCA | 0.204 | 0.150 | 1.364 | 0.175 | (–0.092, 0.501) |

**Note:** HAMD-24 and MoCA were mean‑centred. Low, mean, and high correspond to –1 SD, mean, and +1 SD, respectively. Effect = conditional effect of PHQ-15 on cortisol (simple slope). 95% CI = bootstrap confidence interval (5,000 resamples).
